# Supplementary material for: Phylogenetic diversity and virulence gene characteristics of Escherichia coli from pork and patients with urinary tract infections in Thailand
Source: PLoS One. 2024 Jul 25;19(7):e0307544. doi: 10.1371/journal.pone.0307544 (PMC11271939; doi:10.1371/journal.pone.0307544)
Supplement: S1 Table — (PDF) [file pone.0307544.s001.pdf]

Table S2. PCR primers used in this study.

PCR reaction	Target gene	Primer	Sequence (5'–3')	Amplicon (bp)	T_m (°C)	Reference
**Phylogroups**						
**Quadruplex for typing groups A, B1, B2, D, and F**	*chuA*	chuA.1b	ATGGTACCGGACGAACCAAC	288	59	[1]
		chuA.2	TGCCGCCAGTACCAAAGACA			
	yjaA	yjaA.1b	CAAACGTGAAGTGTCAGGAG	211	59	[1]
		yjaA.2b	AATGCGTTCCTCAACCTGTG			
	TspE4.C2	TspE4C2.1b	CACTATTCGTAAGGTCATCC	152	59	[1]
		TspE4C2.2b	AGTTTATCGCTGCGGGTCGC			
	arpA	AceK.f	AACGCTATTCGCCAGCTTGC	400	59	[1]
		AceK.r	TCTCCCCATACCGTACGCTA			
**Group C**	*trpA*	trpAgpC.1	AGTTTTATGCCCAGTGCGAG	219	59	[1]
		trpAgpC.2	TCTGCGCCGGTCACGCCC			
**Group E**	*arpA*	ArpAgpE.f	GATTCCATCTTGTCAAAATATGCC	301	57	[1]
		ArpAgpE.r	GAAAAGAAAAAGAATTCCCAAGAG			
**Clade 1**	*aes*	aesI.1	CCTCTACTCACCCAAAAGTC	315	49	2]
		aesI.2	ATCACGTAACCACAACGCAC			

Table S1. (continued)

PCR reaction	Target gene	Primer	Sequence (5'–3')	Amplicon (bp)	T_m (°C)	Reference
**Type 1 fimbriae**	*fimH*	fimH-f	AACAGCGATGATTTCCAGTTTGTGTG	465	65	[3]
		fimH-r	ATTGCGTACCAGCATTAGCAATGTCC			
**P fimbriae**	*papC*	pap1	GACGGCTGTACTGCAGGGTGTGGCG	328	65	[4]
		pap2	ATATCCTTTCTGCAGGGATGCAATA			
**S and FIC fimbriae**	*sfa/focDE* region	sfa1	CTCCGGAGAACTGGGTGCATCTTAC	410	65	[4]
		sfa2	CGGAGGAGTAATTACAAACCTGGCA			
**Afa adhesins**	*afaC*	afa-f	CGGCTTTTCTGCTGAACTGGCAGGC	672	65	[4]
		afa-r	CCGTCAGCCCCCACGGCAGACC			
**Hemolysin**	*hlyCA* region	hly-s	AGATTCTTGGGCATGTATCCT	556	65	[5]
		hly-as	TTGCTTTGCAGACTGTAGTGT			
**Cytotoxic necrotizing factor**	*cnf*	cnf-s	TTATATAGTCGTCAAGATGGA	693	58	[3]
		cnf-as	CACTAAGCTTTACAATATTGA			
**Aerobactin**	*iucC*	aer-s	AAACCTGGCTTACGCAACTGT	269	60	[5]
		aer-as	ACCCGTCTGCAAATCATGGAT			

S1 Table. (continued)

PCR reaction	Target gene	Primer	Sequence (5'–3')	Amplicon (bp)	T_m (°C)	Reference
**Virulence genes associated with DEC**						
**STEC**	*stx1*	stx1-F	CTGGATTTAATGTCGCATAGTG	150	55	[6]
		stx1-R	AGAACGCCCACTGAGATCATC			
	stx2	stx2-F	GGCACTGTCTGAAACTGCTCC	255	55	[6]
		stx2-R	TCGCCAGTTATCTGACATTCTG			
**EPEC**	*bfpA*	bfpA-F	AATGGTGCTTGCGCTTGCTGC	324	55	[6]
		bfpA-R	GCCGCTTTATCCAACCTGGTA			
	eae	eae-F	GACCCGGCACAAGCATAAGC	384	55	[6]
		eae-R	CCACCTGCAGCAACAAGAGG			
**ETEC**	*lt*	lt-F	GGCGACAGATTATACCGTGC	450	55	[6]
		lt-R	CGGTCTCTATATTCCCTGTT			
**ETEC**	*st*	st-F	ATTTTTMTTTCTGTATTRTCTT	190	55	[6]
		st-R	CACCCGGTACARGCAGGATT			
**EIEC**	*ial*	ial-F	GGTATGATGATGATGAGTCCA	650	55	[6]
		ial-R	GGAGGCCAACAATTATTTCC			
	ipaH	ipaH-F	GTTCCTTGACCGCCTTTCCGATACCGTC	600	58	[6]
		ipaH-R	GCCGGTCAGCCACCCTCTGAGAGTAC			

S1 Table. (continued)

**PCR reaction**	**Target gene**	**Primer**	**Sequence (5'–3')**	**Amplicon (bp)**	**T_m (°C)**	**Reference**
**EAEC**	*aatA*	CVD432-F	CTGGCGAAAGACTGTATCAT	630	58	[6]
		CVD432-R	CAATGTATAGAAATCCGCTGTT			
**DAEC**	*daaE*	daaE-F	GAACGTTGGTTAATGTGGGGTAA	542	58	[6]
		daaE-R	TATTCACCGGTCGGTTATCAGT			
**Internal control**	16S	16S-F	CCAGCAGCCGCGGTAATACG	996	58	[6]
		16S-R	ATCGGYTACCTTGTTACGACTTC			

UPEC=uropathogenic *Escherichia coli*, DEC=diarrheagenic *E. coli*, STEC= Shiga toxin-producing *E. coli*, EPEC=enteropathogenic *E. coli*, ETEC=enterotoxigenic *E. coli*, EIEC=enteroinvasive *E. coli*, EAEC=enteroaggregative *E. coli*, and DAEC=diffusely adherent *E. coli*

References

1. Clermont O, Christenson JK, Denamur E, Gordon DM. The Clermont *Escherichia coli* phylo-typing method revisited: improvement of specificity and detection of new phylo-groups. Environ Microbiol Rep. 2013;5:58-65. doi: 10.1111/1758-2229.12019.

2. Clermont O, Gordon DM, Brisse S, Walk ST, Denamur E. Characterization of the cryptic *Escherichia* lineages: rapid identification and prevalence. Environ Microbiol. 2011;13:2468-77. doi: 10.1111/j.1462-2920.2011.02519.x.

3. Tarchouna M, Ferjani A, Ben-Selma W, Boukadida J. Distribution of uropathogenic virulence genes in *Escherichia coli* isolated from patients with urinary tract infection. J Glob Infect Dis. 2013;17:e450-e3. doi: 10.1016/j.ijid.2013.01.025.

4. Le Bouguenec C, Archambaud M, Labigne A. Rapid and specific detection of the *pap*, *afa*, and *sfa* adhesin-encoding operons in uropathogenic *Escherichia coli* strains by polymerase chain reaction. J Clin Microbiol. 1992;30:1189-93. doi: 10.1128/jcm.30.5.1189-1193.1992.

5. Usein C-R, Damian M, Tatu-Chitoiu D, Capusa C, Fagaras R, Tudorache D, et al. Prevalence of virulence genes in *Escherichia coli* strains isolated from Romanian adult urinary tract infection cases. J Cell Mol Med. 2001;5:303-10. doi: 10.1111/j.1582-4934.2001.tb00164.x.

6. Fialho OB, de Souza EM, de Borba Dallagassa C, de Oliveira Pedrosa F, Klassen G, Irino K, et al. Detection of diarrheagenic *Escherichia coli* using a two-system multiplex-PCR protocol. J Clin Lab Anal. 2013;27:155-61. doi: 10.1002/jcla.21578.
