# Supplementary material for: Phylogenetic diversity and virulence gene characteristics of Escherichia coli from pork and patients with urinary tract infections in Thailand
Source: PLoS One. 2024 Jul 25;19(7):e0307544. doi: 10.1371/journal.pone.0307544 (PMC11271939; doi:10.1371/journal.pone.0307544)
Supplement: S2 Table — (PDF) [file pone.0307544.s002.pdf]

Table S2. Interpretation of amplified PCR products for phylogenetic groups of *Escherichia coli*.

Phylogenetic group	Gene			
	chuA	*yjaA*	*TSPEA.C2*	*arpA*
**A**	–	–	–	+
	–	+	–	+
**B1**	–	–	+	+
**B2**	+	+	–	–
	+	–	+	–
	+	+	+	–
**A or C**	–	+	–	+
**D or E**	+	–	–	+
	+	–	+	+
**E or clade I**	+	+	–	+
**F**	+	–	–	–
**Clade I**	–	+	–	–
	+	+	–	+
**Clade I or II**	–	+	–	–

Clermont, Christenson (1)

+ =positive

– =negative
